# Glycemic Improvement Using Continuous Glucose Monitoring by Baseline Time in Range: Subgroup Analyses from the DIAMOND Type 1 Diabetes Study

**DOI:** 10.1089/dia.2020.0471

**Published:** 2021-02-25

**Authors:** Peter Calhoun, David Price, Roy W. Beck

**Affiliations:** ^1^ Jaeb Center for Health Research, Tampa, Florida, USA.; ^2^ Dexcom, Inc., San Diego, California, USA.

**Keywords:** Type 1 diabetes, Time in range, Continuous glucose monitoring, Glycemic control

## Abstract

The DIAMOND study demonstrated that real-time continuous glucose monitors (rtCGMs) improve glycemia for adults with type 1 diabetes using multiple daily injections. This analysis explores the relationship between baseline time in range (TIR) and improvement in TIR using rtCGMs or self-monitoring of blood glucose (SMBG). Baseline TIR was divided into three categories: <40% (9.6 h per day), <50% (12 h per day), and <60% (14.4 h per day). Compared with SMBG, use of rtCGMs increased mean TIR by an additional 16 min per day for participants with a baseline TIR <40%, 77 min per day for baseline TIR <50%, and 88 min per day for baseline TIR <60%. A greater percentage of participants increased TIR by >4 h per day using rtCGMs within the three baseline TIR groups. For participants with a baseline TIR <50%, 29% of rtCGM users improved their TIR by >4 h per day compared with no SMBG users (*P* < 0.001). Similar trends were found for improvement in mean glucose and time spent in hyper- and hypoglycemic ranges.

## Introduction

Several studies have shown use of continuous glucose monitors (CGMs) can improve glycemic control and reduce hypoglycemia for participants with type 1 diabetes (T1D).^1–3^ The DIAMOND T1D trial found that participants randomized to receive a real-time continuous glucose monitor (rtCGM) had a 0.6% greater reduction in mean hemoglobin A1c (HbA1c) and spent an additional 77 min per day in range 70–180 mg/dL compared with participants in self-monitoring of blood glucose (SMBG) group.^4^

Beyond the finding of a clinically relevant overall benefit of rtCGMs, it is important to assess improvement at an individual level to identify individuals who can potentially benefit the most from rtCGM use. A secondary analysis of the DIAMOND subject found that improvement in HbA1c was seen within each baseline HbA1c category with the greatest glycemic absolute and relative (vs. SMBG control group) improvement from rtCGM use occurring in participants with a baseline HbA1c >9.0% (Ref.^5^). However, unlike HbA1c, CGM-measured outcomes can assess both short-term and long-term complications. Many researchers have emphasized the importance of time in range (TIR) and hypoglycemia as these represent daily goals for participants with T1D and type 2 diabetes (T2D) and are associated with micro- and macrovascular complications^6–8^ and severe hypoglycemia.^9^ This article extends this analysis by assessing CGM outcomes according to baseline TIR and focusing on individual participant responder outcomes.

## Methods

Details of the protocol and study design have been published elsewhere^4^ and listed on clinicaltrials.gov (NCT02282397). Relevant aspects of the study are summarized herein.

### Study design

The trial was conducted at 24 endocrinology practices across the United States. Major eligibility included age 25 years or older, diagnosis of type 1 diabetes for at least 1 year using multiple daily injections, central laboratory-measured HbA1c level between 7.5% and 10.0%, and no home use of a personal CGM device in the 3 months before the trial. Participants completed a 2-week prerandomization phase using a blinded CGM to assess adherence and establish baseline glycemia. Subjects with at least 85% of CGM data worn at baseline and SMBG testing averaging ≥3 times per day were admitted into the randomization phase.

Participants were randomly assigned to either rtCGM or SMBG group in a 2:1 ratio using a permuted block design stratified by HbA1c level. Participants in the rtCGM group wore a Dexcom G4 Platinum CGM system that measured glucose every 5 min and provided real-time hypoglycemia and hyperglycemia alerts. Participants in the SMBG group performed blood glucose testing ≥4 times per day to manage their glycemia. Clinicians provided general diabetes management education and were able to review downloaded glucose data at follow-up visits to inform treatment recommendations. Follow-up visits occurred at 4, 12, and 24 weeks after randomization. The SMBG group wore a blinded CGM sensor during weeks 12 and 24.

### Statistical methods

CGM outcomes were calculated using the CGM data collected in each group for 7 days during weeks 12 and 24. Participants with CGM data on at least 6 out of the 14 days were included in this analysis. Primary outcome for this analysis was the change in TIR 70–180 mg/dL from baseline to follow-up. Additional outcomes included change in mean glucose, time >180 and 250 mg/dL, time <70 and 54 mg/dL, and glycemic variability as measured by coefficient of variation. Analyses were conducted separately in three baseline TIR categories: <40% (9.6 h per day), <50% (12 h per day), and <60% (14.4 h per day). The proportions of participants increasing their TIR by ≥2 and ≥4 h were tabulated by treatment group in each of these baseline TIR categories.

The estimated mean treatment group difference (rtCGM minus SMBG) for the change in glycemic endpoints was computed using a linear regression model adjusting for baseline glycemic outcome and clinical site as a random effect. The 95% confidence interval for the mean treatment group differences is reported for each continuous outcome. For binary outcomes, Barnard's exact test was used to compare treatment group differences. This test cannot handle the random site effect but yields exact *P*-value calculation for rare events and smaller sample sizes. All *P*-values are two-sided and assessed at the α = 0.05 significance level. For this post hoc analysis, no adjustments were made for multiple hypothesis testing and results are considered exploratory. Analyses were performed using SAS software version 9.4 (SAS Institute, Inc., Cary, NC).

## Results

The DIAMOND trial randomly assigned 158 participants to one of the two treatment arms; 153 participants with T1D (rtCGM, *n* = 101; SMBG, *n* = 52) had at least 6 days of CGM data at baseline and follow-up to be included in this analysis. The demographics of the cohort have been previously reported and are summarized in Supplementary Table S1 for this cohort. At baseline, mean HbA1c was 8.6 ± 0.6% and mean TIR was 46 ± 12% (10.9 ± 2.9 h per day).

**FIG. 1.**
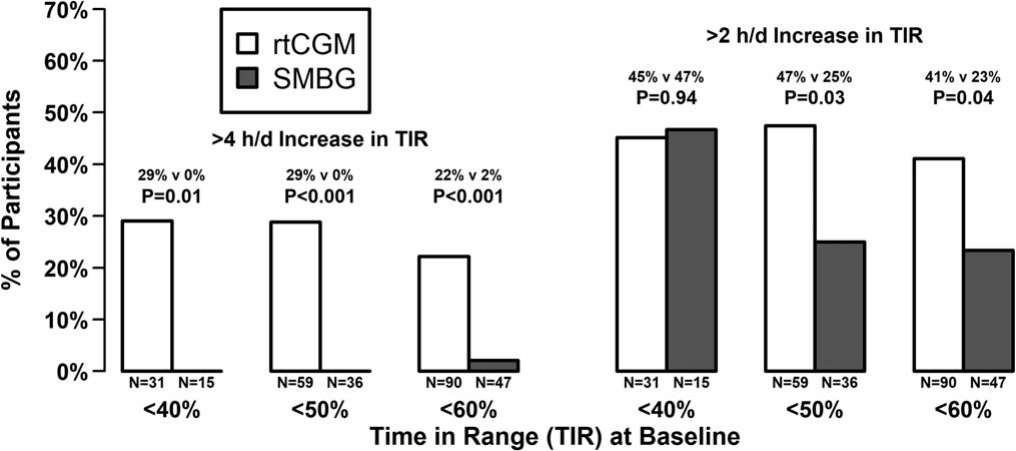
Individual improvement in TIR based on baseline TIR. rtCGM, real-time continuous glucose monitor; TIR, time in range.

Participants with lower baseline TIR had a greater increase in TIR at follow-up (Supplementary Fig. S1). Mean increase in TIR was greater among rtCGM users versus SMBG users for all three baseline categories (16 min per day for baseline TIR <40%, 77 min per day for baseline TIR <50%, and 88 min per day for baseline TIR <60%; Table 1). A greater percentage of participants using rtCGM had >4 h per day within the three baseline TIR groups with the most noticeable difference occurring for participants with a baseline TIR <50% (12 h per day) with 29% of rtCGM users improving their TIR by >4 h per day compared with no SMBG users (*P* < 0.001; Fig. 1). Similarly, these participants were also more likely to have >2 h per day increase in TIR using the rtCGM compared with SMBG (47% rtCGM users vs. 25% SMBG users, *P* = 0.03).

**Table 1.** Treatment Effect Sizes Based on Baseline Time in Range

	Baseline TIR		
	<40% (*N* = 46)	<50% (*N* = 95)	<60% (*N* = 137)
CGM metric	Treatment effect size^a^		
TIR (min per day)	16 (−69, 101)	77 (15, 140)	88 (32, 143)
Mean glucose (mg/dL)	−3 (−17, 12)	−10 (−19, 0)	−11 (−19, −3)
Time above 180 mg/dL (min per day)	−5 (−109, 99)	−59 (−126, 8)	−70 (−130, −10)
Time above 250 mg/dL (min per day)	−23 (−111, 65)	−76 (−135, −18)	−89 (−136, −42)
Time below 70 mg/dL (min per day)	−9 (−28, 11)	−17 (−35, 1)	−21 (−35, −8)
Time below 54 mg/dL (min per day)	−7 (−19, 5)	−12 (−21, −2)	−15 (−22, −7)

^a^ Treatment effect size is the difference between the mean outcome in the rtCGM group minus the mean outcome in SMBG group controlling for outcome at baseline and a random site effect. Values are reported as point estimate (95% CI).

CGM, continuous glucose monitor; CI, confidence interval; rtCGM, real-time CGM; SMBG, self-monitoring of blood glucose; TIR, time in range.

Time spent in hypoglycemia decreased during follow-up for both treatment groups (Supplementary Table S2). The use of rtCGMs decreased time spent <70 mg/dL by an estimated 9, 17, and 21 min per day for participants with a baseline TIR <40%, <50%, and <60%, respectively (Table 1). Mean time spent <54 mg/dL was significantly lower in the rtCGM group for participants with a baseline TIR <50% or <60%. Participants in the rtCGM group with a baseline TIR <60% also reduced their mean glucose and time spent in hyperglycemia significantly more than participants in SMBG group.

## Discussion

Use of rtCGMs increased TIR and lowered time spent in hypoglycemia compared with SMBG use. Participants with a baseline TIR <40% (9.6 h per day) experienced the greatest improvement during follow-up (117 min per day mean increase in rtCGM; 99 min per day mean increase in SMBG), but also had the smallest difference between rtCGM and SMBG compared with other baseline TIR categories. In this group of participants with low TIR at baseline, the improved glycemia may be due to regression to the mean and an overall study effect. Use of rtCGMs added an additional improvement by increasing the mean time by 16 min per day, although this was not statistically significant. For participants with worse glycemic control, the rtCGM effect size on TIR differed from HbA1c, where Billings et al. found DIAMOND participants with an HbA1c ≥9.0% had the greatest improvement in HbA1c *and* the largest rtCGM effect sizes.^5^ We conjecture this discrepancy could be attributed to the greater variability with TIR at baseline (only 2 weeks of CGM data) and the small sample size of 15 SMBG users with a baseline TIR <40% who happened to have a large increase in TIR.

The mean increase in TIR represents a clinically meaningful outcome, but it can be misleading to equate a mean effect size with an individual effect size. Although rtCGMs only increased the TIR by an extra 16 min per day compared with SMBG in the participants with low baseline TIR, participants improved their TIR substantially in both glucose monitoring groups. However, there is a greater chance of increasing their TIR by >4 h in the rtCGM group compared with SMBG group (29% vs. 0%; *P* = 0.01). This responder analysis compares individual improvement by the glucose monitoring method.

There has been a recent push for responder analyses with the recent consensus article describing specific glycemic targets for individuals with diabetes.^10^ Assessing the proportion of individuals meeting these targets is helpful in the interpretation in addition to figures showing the distribution of glycemia for the treatment arms. However, assessing individual change from baseline may be a better target metric if baseline glycemia is poor and there are too few participants achieving the individual targets. The DIAMOND T1D study included HbA1c responder outcomes as secondary endpoints such as the percentage of participants with an HbA1c <7.0%, a relative reduction ≥10%, an absolute reduction ≥1%, and either an absolute reduction ≥1% or an HbA1c <7.0%. Participants with low baseline HbA1c will have a greater chance meeting the HbA1c target of 7.0%, but a lower chance of reducing their HbA1c by at least 1%; evaluating these outcomes separately and combined can be useful when assessing a treatment effect. Similarly, defining targets for change of CGM-measured outcomes can offer a different perspective not directly seen in conventional statistical analyses. CGM-measured targets could also include composite metrics such as achieving a euglycemic target without increasing hypoglycemia.

This analysis demonstrated that rtCGM use helps many participants achieve increases of TIR of 2 or more hours daily and some participants reach a remarkable increase of at least 4 h daily spent in target range. This increase in TIR occurred while significantly reducing hypoglycemia for patients with a baseline TIR <60%. This study used an older Dexcom G4 Platinum CGM, but we expect the current generation (Dexcom G6) to obtain the same or greater benefits with respect to increasing TIR and reducing hypoglycemia.^11^

## Conclusion

Although HbA1c is currently the accepted metric for assessing the efficacy of diabetes products, guiding medication adjustments, and supporting regulatory approval and reimbursement policies, it has limitations^12^ and TIR is now recognized as a key metric of glycemic control. TIR was rated as having a major impact on the quality of daily life in a large survey of people living with TID and T2D.^13^ TIR was found to be the optimal percentage metric to discriminate between different subjects^14^ and has been associated with diabetic complications. In many study participants who were poorly controlled with TIR below the consensus TIR target, use of rtCGM had substantial glycemic benefits. The increase in TIR observed in many of these study participants may result in quality of life and health benefits.^15^ TIR is a clinically meaningful metric that can proficiently evaluate the effectiveness of a treatment.

## Supplementary Material

Supplemental data

Supplemental data

Supplemental data
