# Supplementary material for: Glycemic Improvement Using Continuous Glucose Monitoring by Baseline Time in Range: Subgroup Analyses from the DIAMOND Type 1 Diabetes Study
Source: Diabetes Technol Ther. 2021 Feb 25;23(3):230–3. doi: 10.1089/dia.2020.0471 (PMC7906860; doi:10.1089/dia.2020.0471)
Supplement: Supplemental data [file Supp_TableS1.docx]

# Supplemental Table S1. Baseline Characteristics and Glycemia of the Analysis Cohorts.

|  | **Overall**  **(N=153)** | **rtCGM**  **(N=101)** | **SMBG**  **(N=52)** |
| --- | --- | --- | --- |
| **Baseline Characteristics** |  |  |  |
| Age (years) *mean ± SD* | 48 ± 13 | 46 ± 14 | 51 ± 11 |
| Gender – Female *n (%)* | 67 (44%) | 45 (45%) | 22 (42%) |
| Diabetes Duration (years) *median (IQR)* | 19 (10-31) | 19 (9-30) | 19 (11-35) |
| Education *n (%)* |  |  |  |
| Less than bachelor’s degree | 69 (46%) | 44 (46%) | 22 (43%) |
| Bachelor’s degree | 62 (41%) | 42 (44%) | 19 (37%) |
| Graduate/professional degree | 20 (13%) | 10 (10%) | 10 (20%) |
| Body Mass Index (kg/m^2^) *mean ± SD* | 28 ± 5 | 28 ± 6 | 27 ± 4 |
| Self-reported number of blood glucose tests per day *mean ± SD* | 3.9 ± 1.4 | 3.9 ± 1.3 | 4.1 ± 1.6 |
| Total daily insulin dose (U/kg/d) *median (IQR)* | 0.7 (0.5-0.9) | 0.7 (0.5-0.9) | 0.6 (0.5-0.9) |
| **Baseline Glycemia** |  |  |  |
| HbA1c (%) *mean ± SD* | 8.6 ± 0.6 | 8.6 ± 0.6 | 8.6 ± 0.6 |
| Mean Glucose (mg/dL) *mean ± SD* | 187 ± 27 | 187 ± 26 | 186 ± 30 |
| Time in Range (h/day) *mean ± SD* | 10.9 ± 2.9 | 11.0 ± 3.0 | 10.9 ± 2.9 |
