# Supplementary material for: Glycemic Improvement Using Continuous Glucose Monitoring by Baseline Time in Range: Subgroup Analyses from the DIAMOND Type 1 Diabetes Study
Source: Diabetes Technol Ther. 2021 Feb 25;23(3):230–3. doi: 10.1089/dia.2020.0471 (PMC7906860; doi:10.1089/dia.2020.0471)

# Supplemental Figure S1. Change in TIR by baseline TIR. Each dot represents one participant. Correlation, r, between baseline TIR and change in TIR reported by treatment group.


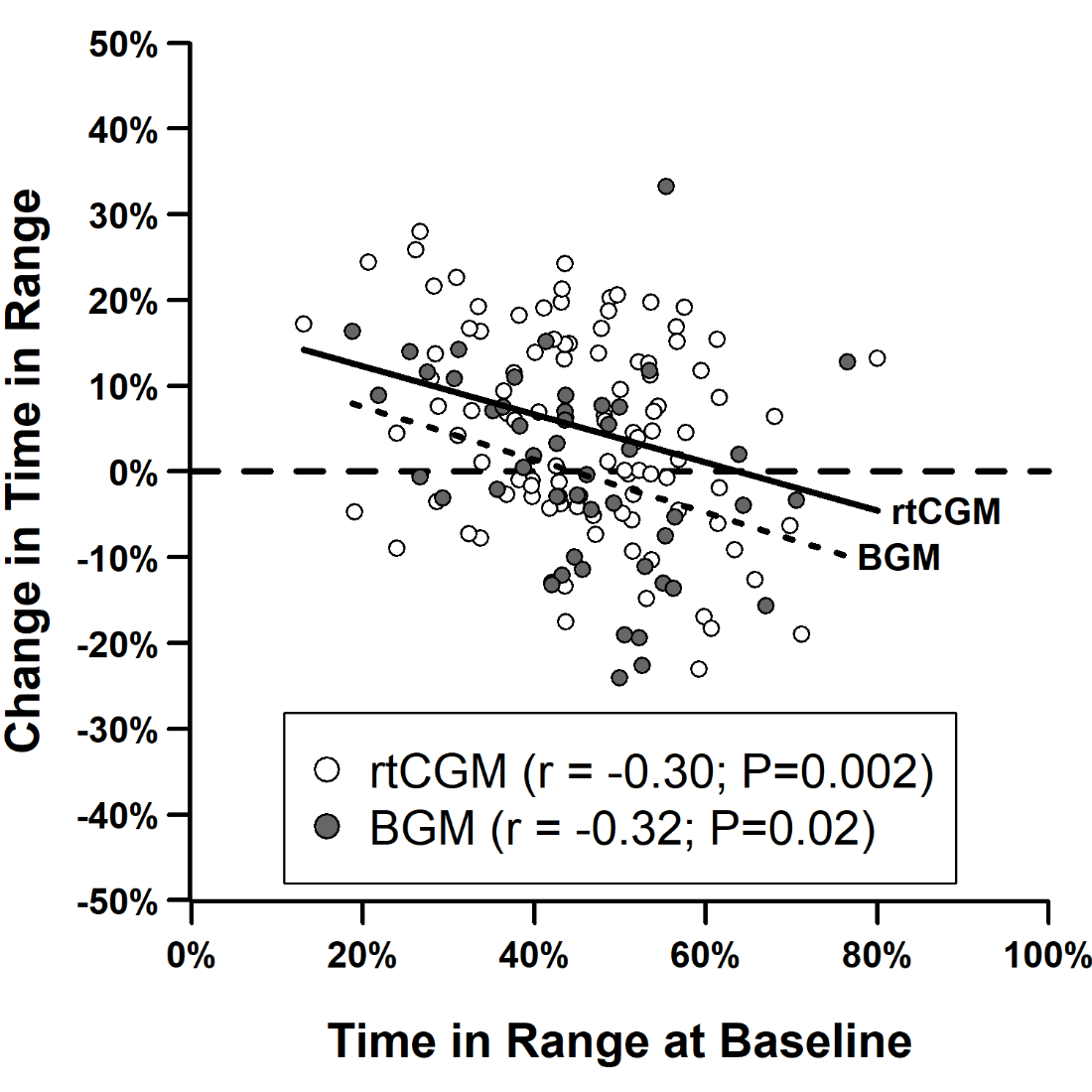

Supplement: Supplemental data [file Supp_FigS1.docx]
