# Supplementary material for: Glycemic Improvement Using Continuous Glucose Monitoring by Baseline Time in Range: Subgroup Analyses from the DIAMOND Type 1 Diabetes Study
Source: Diabetes Technol Ther. 2021 Feb 25;23(3):230–3. doi: 10.1089/dia.2020.0471 (PMC7906860; doi:10.1089/dia.2020.0471)
Supplement: Supplemental data [file Supp_TableS2.docx]

Supplemental Table S2. Change in CGM-measured glycemia by treatment group.

|  | Baseline TIR | | | | | |
| --- | --- | --- | --- | --- | --- | --- |
|  | <40% | | <50% | | <60% | |
|  | rtCGM  (N=31) | SMBG  (N=15) | rtCGM  (N=59) | SMBG  (N=36) | rtCGM  (N=90) | SMBG  (N=47) |
| CGM Metric | Change in CGM metric ^1^ | | | | | |
| Time in range (min/day) | 102  (-24, 248) | 108  (7, 167) | 102  (-24, 248) | 37  (-44, 119) | 88  (-38, 218) | -5  (-144, 111) |
| Mean glucose (mg/dL) | -7  (-34, 1) | -21  (-32, 7) | -9  (-31, 1) | 3  (-20, 11) | -6  (-25, 7) | 3  (-18, 16) |
| Time above 180 mg/dL (min/day) | -90  (-284, 52) | -127  (-173, 13) | -90  (-257, 52) | -26  (-130, 51) | -51  (-208, 53) | 13  (-119, 125) |
| Time above 250 mg/dL (min/day) | -75  (-225, 5) | -100  (-230, 24) | -76  (-191, 5) | 18  (-107, 75) | -63  (-148, 25) | 24  (-100, 123) |
| Time below 70 mg/dL (min/day) | -1  (-33, 17) | -2  (-17, 28) | -10  (-33, 5) | 0  (-16, 24) | -15  (-44, 4) | -2  (-34, 24) |
| Time below 54 mg/dL (min/day) | -6  (-15, 7) | 0  (-7, 10) | -6  (-20, 5) | 0  (-9, 14) | -9  (-26, 2) | 0  (-12, 16) |

^1^ Change in CGM metric is the difference between follow-up and baseline. Values are reported as Median (quartiles).
